# Recent advances in understanding and managing liver transplantation

**DOI:** 10.12688/f1000research.8768.1

**Published:** 2016-12-21

**Authors:** Francesco Paolo Russo, Alberto Ferrarese, Alberto Zanetto

**Affiliations:** 1 Gastroenterology/Multivisceral Transplant Unit, Department of Surgery, Oncology and Gastroenterology, Padua University Hospital, Padua, Italy

**Keywords:** liver transplantation, liver disease, liver donors, living donor liver transplantation, chronic liver diseases, liver failure

## Abstract

Liver transplantation (LT) has been established as the most effective treatment modality for end-stage liver disease over the last few decades. Currently, patient and graft survival after LT are excellent, with 1- and 5-year survival of 90% and 80%, respectively. However, the timing of referral to LT is crucial for improving survival benefit and outcome. The current shortage of donors and the increasing demand for LT currently lengthen the waiting time. Thus, waiting list mortality is about 10–15%, according to the geographical area. For this reason, over the last several years, alternatives to deceased donor LT and new options for prioritizing patients on the waiting list have been proposed.

## Introduction

The continuous improvement of surgical techniques, organ conservation, and immunosuppression management as well as optimization of intensive care have improved the results of liver transplantation (LT), and today this surgical procedure is a viable treatment option for patients with end-stage liver disease or acute liver failure (ALF).

Between 1963 and 1968, Thomas Starzl and Roy Calne performed the first LT in Denver (USA) and Cambridge (Europe), respectively ^1^. Now, LT is the standard treatment for ALF and chronic liver failure of all etiologies, and over 80,000 procedures have been carried out since its inception. Survival rates are significantly better now than they were a quarter of a century ago: 96% at 1 year and 71% at 10 years post-LT ^2^. This review focuses on hot topics in the field of transplant hepatology and includes indications for LT, timing for LT, use of extended criteria donors, management of early and long-term complications after LT, and transplant benefit.

## Enlarging the donor pool

Since indications for LT are increasing, transplant teams are searching for new ways to increase the donor pool. In the United States, less than 40% of patients on the waiting list eventually receive a graft, and almost 10% die while waiting owing to a paucity of organs compared to the need for organs for transplantation ^3^. For this reason, previous and strict criteria for accepting organs for liver donation have slowly become more liberal ^4^.

### Extended criteria donors

Although the definition of an extended donor has not been thoroughly established, most agree that it conveys a higher risk of either physiologic dysfunction or infectious/metabolic disease transmission. Extended criteria can be separated into two groups: donor-related risk factors and surgical technique-related issues. Donor-related issues include donation after cardiac death (DCD), advanced age, increased cold ischemia time, ABO incompatibility, steatosis, previous malignancies in the donor, hepatitis C virus (HCV) or hepatitis B virus (HBV) infection, human T-cell lymphotrophic virus type I/II infection, or other active infections. These extended criteria donors can generally be accepted or declined by the transplant team during evaluation of the allograft. Surgical technique-related issues of extended donors include split LT (SLT) and partial grafts used in living donor LT (LDLT). Both of these methods can provide a graft when a whole cadaveric organ is unavailable ^5^. In the first group, a special mention needs to be made for DCD, whereas amongst the latter we will address the SLT procedure and LDLT.

***Donation after cardiac death.*** DCD organs are expected to expand the donor pool. Indeed, in the past 10 years in the US, 2,710 liver donors have been DCD organ donors, with the largest numbers used in the last 2 years ^6^. Although previous experiences with DCD have been associated with greater risk of graft failure ^7^, more recent reports did not confirm this finding, showing similar outcomes between DCD and donation after brain death (DBD) ^8^. Still, some limitations need to be considered by the transplant team. In order to ensure a similar outcome to those associated with DBD, several variables need to be taken into account, such as donor age, warm and cold ischemia times, and duration of donor hypotension or hypoxemia ^9– 11^. Hopefully, in the next few years, improving our ability to perform ante mortem interventions will improve the likelihood of successful donation and graft outcomes. On the other hand, *post mortem* intervention such as the use of machine perfusion (MP) systems to improve graft function during the preservation period will be an additional challenging issue to improve DCD utilization. MP indicates several dynamic strategies applied *ex vivo* of organs for transplantation that aimed to improve the static cold storage preservation ^12^. Recently, De Carlis *et al*. ^13^ reported their experience in using the MP in the setting of DCD. Even if the relatively small sample size (7 cases) limits the general applicability of the results, patient and graft survival were both 100% after a mean follow up of 6.1 months (range 3 – 9) and no cases of ischemic cholangiopathy occurred during the follow-up. Hopefully, the extensive use of MP will lead to a significant increase of the availability of transplant livers as well as a significant reduction in several types of graft dysfunction and biliary complications.

***Split liver transplantation.*** Over the 25 years since the first LTs were performed ^14^, in light of continuing organ shortages and growing numbers of patients dying whilst waiting for transplants, SLT enlarges the donor pool and is one of the few surgical options to do so. LT performed with split grafts in Europe and in the US accounted for about 6% in the past decade ^15^. Although the outcomes of LTs performed using partial grafts are good, there are specific complications associated with this technique. For example, small for size syndrome, related to a reduced ratio between graft and recipient body weight, is characterized by prolonged jaundice, graft dysfunction, and sometimes graft failure. Favorable results with SLT depend on not only the technical factors but also scrupulous recipient and donor selection.

***Living donor liver transplantation.*** LDLT has emerged as a promising alternative to overcome donor shortage ^16^. The improvements in LDLT have led to the expansion of the recipient criteria to include patients previously considered not suitable for LT because of older age or co-morbidities. Living donors older than 45 years are often discarded, since the risks of these LDLTs remain controversial. Goldaracena *et al*. ^17^ compared patients receiving a LDLT from 91 donors aged ≥50 years with 378 younger than 50 years. The incidence of biliary complications as well as graft and patient survival at 1, 5, and 10 years were similar between both groups. Similarly, Oezcelik *et al*. ^18^ evaluated the use of LDLT in recipients older than 70 years. No significant differences in complications, hospital stay, perioperative mortality, or median survival compared to the younger group were found. Although LDLT is not a “100%” safe procedure and donor death rate has been reported around 0.1–0.3% (possibly reaching 0.5% when using the right hemiliver for adult-to-adult transplantation) ^19^, the understanding of the biochemical mechanisms of graft injury and the possibility of promoting liver regeneration will be the key issues for the improvement of the use of partial liver grafts.

## Donor-recipient matching

Understanding the interactions among donor, graft, and recipient factors will ensure the best outcomes are attained after LT. Donor-recipient (D-R) matching can be defined as “the technique to check D-R pairs adequately associated by the presence of the constituents of some patterns from donor and patient variables” ^20^. Several factors play a role in this scenario, and four different categories should be considered at least: the donor’s age, gender, ethnicity, and viral serology; the graft’s size and quality; the recipient’s age, size, and gender; and the transplant’s major or minor blood group compatibility as well as immunological factors ^21^. A detailed analysis of all these factors goes much further than the scope of this review. However, in the last few years, it has become clear that suitable matching together with adjusting surgical practise and developing novel peri-transplant approaches enables the utilization of grafts that would normally be rejected, thereby widening the donor pool. It is our opinion that they should include not only “simple mathematical variables” but also, at the same time, the global probability of death whilst waiting for transplant, survival after transplantation, cost-effectiveness, and survival benefit. We can achieve transparency, justice, utility, and equity when we consider all factors in one method ^20^.

## Indications for liver transplantation

Candidates for LT must have irreversible ALF, progressive end-stage liver disease, or rarer diseases characterized by a normal liver producing toxic products (i.e. urea cycle defects, familial amyloidosis, hyperoxaluria glycogenosis, and low-density lipoprotein [LDL] receptor defects). LT should be considered for any patient in whom survival after LT will exceed life expectancy of the underlying disease or where a significant increase in quality of life can be achieved. In more detail, intractable pruritus in cholestatic liver disease recipients, abdominal pain due to polycystic liver disease, and persistent/refractory hepatic encephalopathy are clinical conditions that severely affect patients' and relatives' quality of life; thus, they are considered to be accepted indications for LT ^22, 23^.

Although HCV is currently the leading etiology among adult LT recipients ^24^, in 2012 non-alcoholic steatohepatitis (NASH) was the etiology with the most rapid rise in frequency, increasing 4-fold from 2002 to 2012 ^25^. Other main indications for LT are alcoholic liver disease, HBV-related cirrhosis, primary sclerosing cholangitis (PSC), primary biliary cholangitis (PBC), and autoimmune hepatitis (AIH). Liver malignancies, like intra-hepatic hepatocellular carcinoma (HCC) or other rarer benign and malignant tumors, are considered to be a common indication for LT. Biliary atresia, Alagille syndrome, and metabolic liver diseases are the most common indications in the pediatric population ^23^.

Acute alcoholic hepatitis (AAH) is associated with 1, 3 and 6 months mortality of 16%, 27%, and 40% respectively ^26^. Steroids are accepted treatment for severe AAH although their use is still a matter of debate. A meta-analysis by Rambaldi *et al*. ^27^, including 721 patients, showed that steroids did not reduce mortality compared with placebo or no intervention. In a very select group of patients with AAH not responding to steroids, LT has been experimentally proposed after a selection process based on a multidisciplinary approach that involves transplant hepatologist, anesthetist, surgeon, ethicist, psychiatric and nurse ^28^. The 6 and 24 months survival were significantly higher than not transplanted matched controls (77% vs 23%). Furthermore, the risk of recidivism was about 11.5% up to more than 3 years after LT, not significantly higher than the risk of patients who were transplanted after 6 month of abstinence.

There are also absolute and relative contraindications for LT, such as active alcohol and illicit drug abuse, extrahepatic malignancies (including extrahepatic HCC or neoplastic portal vein thrombosis), sepsis, severe pulmonary hypertension, coexistent medical disorders (mainly cardiopulmonary diseases or neurological organic diseases), and poor familial/social support. Relative contraindications include recipient age over 70 years, severe malnutrition or morbid obesity (BMI <18 or >40, respectively), severe osteoporosis with spontaneous fracture, cholangiocarcinoma, and previous extensive abdominal surgery.

## Timing to liver transplantation

### Chronic liver diseases

During the assessment of a patient with liver disease, signs of decompensation (jaundice, moderate to severe ascites, previous variceal hemorrhage, or hepatic encephalopathy), suggest the need for a referral for evaluation for LT ^29^.

The ideal timing for performing LT should be balanced between mortality rate while on the waiting list and perioperative and postoperative mortality (10–15% at 1 year and 15–25% at 3 years) ^30^. This decision must take into account both quality of life and prognosis related to natural history of liver disease as well as post-surgical mortality and morbidity. For these reasons, patients should be strictly selected and prioritized using prognostic scores. Currently, severity and mortality with liver disease is best highlighted by the MELD (model for end-stage liver disease) score, which predicts a recipient’s survival within 6 months through a logarithmic scale that includes as the variables a patients total bilirubin, INR, and creatinine.

However, the MELD score without modification was shown not to adequately reflect all of the complications of portal hypertension (e.g. hepatic encephalopathy and severe ascites). Thus, some efforts to update the prognostic value of the MELD score have been made over time. The MELD-Na formula (which added serum sodium to the abovementioned biochemical parameters) ^31^ is now the most used formula to predict survival among candidates for LT, especially for those with significant portal hypertension.

The MELD-based model applied a "sickest-first policy" and was adopted for use for graft allocation in US by UNOS in 2002 and in Europe by Eurotransplant in 2007. In a large study, LT candidates with a MELD score ≥18 demonstrated considerable transplant benefit, while those transplanted with a MELD score <15 showed a higher mortality rate when compared to those still waiting for transplant ^32^.

### Hepatocellular carcinoma

HCC is one of the common indications for LT worldwide. About 20 years ago, the Milan criteria (MC; three nodules <3 cm or a single nodule <5 cm without vascular invasion) were proposed to select patients with HCC achieving the best survival after LT. Patients within the MC had a 5-year survival of about 70% with a tumor recurrence of <10% ^33^. This survival matches the post-transplant survival of most other indications for LT; therefore, the MC still represent the globally accepted score system to consider patients with HCC suitable for LT. However, modest expansion of the MC could increase the number of selected candidates for LT without negative impact on survival ^34, 35^. Other authors recently proposed the evaluation of tumor behavior as a criterion for listing ^36^. Even though the MC remain the preferred model to allocate organs to patients with HCC, because of the excellent post-LT survival, a modest expansion of these criteria could be one of the future challenges for transplant hepatologists in the next decade ^37^.

### Acute liver failure

ALF is a clinical manifestation of sudden and severe hepatic injury, mainly characterized by the onset of hepatic encephalopathy and severe coagulopathy ^38^, being caused by a massive necrosis of liver parenchyma exceeding the so-called minimum "critical mass" of hepatocytes capable of preserving organ function ^39^. The most important etiological factors of ALF are viral infection (mainly HBV), drugs (mainly acetaminophen but also herbal compounds, non-steroidal anti-inflammatory drugs, antibiotics, and statins). Other less common causes are severe AIH, Wilson’s disease, mushroom poisoning, and toxic substance consumption (e.g. ecstasy, MDMA, and cocaine). In a significant percentage of patients, no etiological factor can be found, especially in the pediatric population ^40^. The timing of encephalopathy onset is crucial to establishing the type of ALF (e.g. hyper acute, acute, or subacute) and to providing an adequate short-term prognosis. Different selection criteria for emergency LT are used worldwide. Commonly used criteria evaluate multiorgan impairment (encephalopathy and metabolic acidosis), etiology, and severity of coagulopathy as the most important factors for listing ALF patients. King's College criteria ^41^ are the most commonly applied; however, other algorithms have been proposed over time ^42, 43^. Nevertheless, several factors, such as recipient age, severity of pre-transplant illness, comorbidities, and the nature of graft used, could affect the outcome of emergency LT ^38^.

## Transplant benefit

It is important to determine, in every aspect of medicine, whether or not an administered therapy will provide benefit to the patient. When dealing with organ failure, not all patients can be given the ideal treatment (organ transplantation) because of low availability. In some situations, even in the event of there being enough donor organs, for some patients the benefit of LT is not enough if compared to the waiting list mortality and the high postoperative and perioperative mortality, according to the literature. In LT programs, organ allocation reflects the policy of the “sickest first”: the organ is assigned to the recipient with the highest MELD score. In addition, the organ allocation policy can also be based on the transplant benefit, which is the benefit that best balances the recipient’s urgent need for transplantation with the need to optimize resource donation, producing good postoperative results in terms of patient and graft survival ^44, 45^.

## Early post-transplant and long-term follow-up

Of the life-threatening complications related to LT, most occur perioperatively. These include primary graft dysfunction, acute rejection episodes, severe infections, and technical complications such as hepatic artery thrombosis or biliary leaks ^46^. Conversely, post-LT long-term morbidity and mortality is caused mainly by the adverse effects of the immunosuppressive drugs. Acute rejection occurs with higher incidence within 2 weeks after transplantation, with a prevalence of 25–60% within 12 months after LT; acute rejection is treatable with steroid therapy or with more potent immunosuppressive drugs.

Chronic rejection occurs in about 5% of cases, usually after 6 months, and may evolve irreversibly with end-stage liver disease. Even if the rate of graft loss due to chronic rejection has significantly decreased to less than 2%, re-LT is indicated in those non-responders to medical therapy ^47^.

Finally, the cornerstone of LT recipients’ long-term management comprises not only the preservation of graft function but also the prevention and treatment of metabolic complications and cardiovascular disease, as well as regular screening for malignancies ^48^.

## Conclusion

LT is the treatment of choice for selected patients with end-stage liver disease, with HCC within restricted criteria, or with ALF. Graft and patient survival are markedly improving compared to the early period of LT owing to greater expertise in the surgical procedure and management of immunosuppressive therapy.

An important limitation to LT is donor shortage. Split liver and the use of organs from living donors, extended criteria donors, or DCD are techniques used to increase the number of transplants. Optimizing the donor pool while offering equal access to LT have become the main challenges today. Patient survival is now >90% and 70–80% at 1 and 5 years, respectively. However, there is a need to identify which patients achieve significant survival benefit from transplantation and which do not so that resources are better directed to achieve greater good for all patients with liver disease.

## Abbreviations

AIH, autoimmune hepatitis; ALF, acute liver failure; DBD, donation after brain death; DCD, donation after cardiac death; D-R, donor-recipient; HBV, hepatitis B virus; HCC, hepatocellular carcinoma; HCV, hepatitis C virus; LDLT, living donor liver transplantation; LT, liver transplantation; MC, Milan criteria; MELD, model for end-stage liver disease; SLT, split liver transplantation.
